# Gorlin-Goltz-Syndrom – nicht nur ein Syndrom maligner Lidtumoren

**DOI:** 10.1007/s00347-021-01371-y

**Published:** 2021-03-26

**Authors:** C. Kortuem, A. Abaza, C. Schramm, F. Kortuem

**Affiliations:** Universitätsklinikum Tübingen, Departement für Augenheilkunde, Eberhard Karls Universität Tübingen, Elfriede-Aulhorn-Str. 7, 72076 Tübingen, Deutschland

## Anamnese

Die Überweisung einer 21-jährigen Patientin Ende 2020 erfolgte zur Mitbeurteilung bei Verdacht auf rezidivierende Hordeola. Die Patientin beklagte unscharfes Sehen durch rezidivierende, spontane, eitrige Sekretentleerungen und Druckschmerzen sowie Fremdkörpergefühl seit 3 Jahren. Weitere Symptome, insbesondere eine Rötung der Bindehaut, wurden nicht beklagt. Teilweise würden sich die Zysten auch spontan zurückbilden. Anamnestisch waren an beiden Augen Exzisionen von Hordeola bereits 2017 und 2018 erfolgt. In der Vergangenheit seien wegen der Zysten Dexa-Gentamicin-Augensalbe und -Augentropfen in verschiedenen Dosierungen und teilweise über Wochen angewendet worden, zuletzt im August 2020. Die Zysten seien jedoch durch die genannte Therapie nicht regredient gewesen. Aktuell würde 1‑mal pro Tag beidseits dünnflüssiges Tränenersatzmittel angewendet.

In der Allgemeinanamnese war ein Gorlin-Goltz-Syndrom (GGS) bekannt, dessen heterozygote Neumutation im *PTCH1*-Gen bestätigt worden war, und sich bei der Patientin durch multiple Kiefer- und Epidermalzysten sowie eine grenzwertige Makrozephalie und Ohrdysmorphie äußerte. Die Kieferzysten wurden bereits in 7 Operationen seit dem 13. Lebensjahr exzidiert. Zudem wurden Zysten in den Nasennebenhöhlen sowie an der Vulva operativ entfernt. Medikamentös wurde die Patientin mit L‑Thyroxin bei Hypothyreose behandelt. Ophthalmologisch war die Anamnese außer der oben beschriebenen Symptomatik blande, insbesondere wurden bisher keine Basalzellkarzinome diagnostiziert.

## Befund

**Figure 1:**
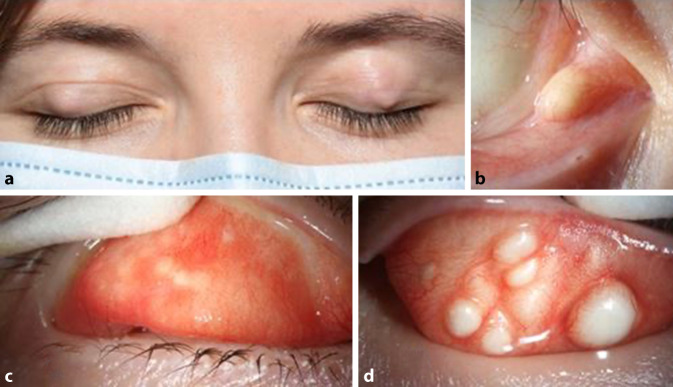

Es zeigten sich links mehr als rechts multiple, nicht verschiebliche, zystische, gelblich-weiße Prominenzen subtarsal oben (Abb. 1) und derbe, rundliche, gut abgrenzbare Verdickungen, die außen am Oberlid tastbar waren. Zudem bestand eine gut abgrenzbare, zystische Raumforderung der Bindehaut temporal unterhalb der rechten Karunkel, welche die gleiche Farbe aufwies wie die subtarsalen Prominenzen. Der bestkorrigierte Visus (sphärisches Äquivalent beidseits −1,25 dpt) war voll. Der intraokulare Druck betrug rechts 16 mm Hg und links 14 mm Hg. Der sonstige ophthalmologische Untersuchungsbefund der vorderen und hinteren Augenabschnitte war unauffällig. Insbesondere bestanden keine Katarakt, Kolobome oder ein Mikrophthalmus.

**Figure 2:**
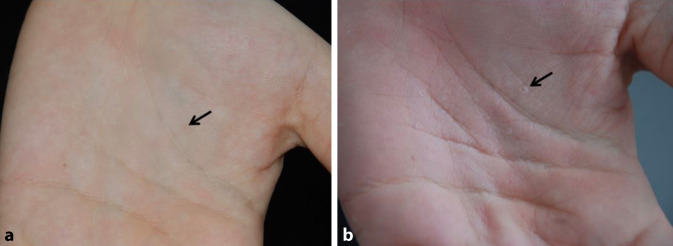

Nebenbefundlich zeigten sich sog. „Pits“ an beiden Händen palmarseitig (Abb. 2). Dabei handelte es sich um rundliche, erhabene hyperkeratotische Läsionen von maximal 2 mm Durchmesser mit einer kleinen, zentralen Verdunkelung. Bei guter Befeuchtung von mehreren Minuten mit Leitungswasser schwollen diese an und waren besser sichtbar.

## Diagnose

Beidseitige multiple intratarsale Keratinzysten, ausgehend von den Meibom-Drüsen der Oberlider, sowie eine rechtsseitige Keratinzyste in der bulbären Bindehaut im Rahmen des Gorlin-Goltz-Syndroms.

## Therapie und Verlauf

Da die Patientin über rezidivierende, sehbeeinträchtigende Sekretabsonderungen klagte, wurde eine erneute Inzision mit Kürettage angeboten. Bis zum Operationstermin 5 Wochen nach der Erstvorstellung hatte sich die Zyste temporal unterhalb der rechten Karunkel spontan entleert und war nicht mehr sichtbar. Intraoperativ wurden die Zysten subtarsal mit dem Skalpell inzidiert, und es entleerte sich spontan viel talgartiges Material. Nach Exprimation des Sekretes zeigten sich im Tarsus harte Zystenkapseln, welche mit dem scharfen Löffel kürettiert wurden. Da die Kapseln mit dem Tarsus verklebt waren, konnten sie ohne Tarsektomie nicht vollständig exzidiert werden, sondern wurden zusätzlich elektrokoaguliert, um ein Rezidiv zu vermeiden. Am Ende der Operation wurde Dexa-Gentamicin-Augensalbe verabreicht. Zudem wurde empfohlen, die Salbe 2 Wochen postoperativ 2‑malig pro Tag anzuwenden, um einer Infektion und Vernarbung vorzubeugen und die postoperative Schwellung zu reduzieren. In der histologischen Untersuchung konnten entsprechend der fehlenden Exzisionsmöglichkeit einer Zystenwand beidseits nur Hornlamellen nachgewiesen werden, die histologisch auch in den, für das Krankheitsbild typischen, odontogenen Keratozysten im Kiefer nachgewiesen werden können. Zehn Tage postoperativ berichtete die Patientin, dass die Bindehautzyste am Rand der rechten Karunkel erneut auffällig geworden sei. Es bestünde eine beidseitige deutliche Oberlidschwellung. Bei deutlicher Lichtempfindlichkeit trug die Patientin bis dahin eine Sonnenbrille. Die Sekretabsonderungen hatten sich verbessert. Bei einem erneuten Telefonat ca. 7 Wochen postoperativ beschrieb die Patientin, dass sich am temporalen rechten Oberlid eine neue Zyste gebildet habe. Da keine funktionellen Einschränkungen bestanden, wünschte die Patientin vorerst keinen weiteren Eingriff.

## Diskussion

Das GGS oder auch Basalzell-Nävus-Syndrom gehört mit einer Prävalenz von 1–9/100.000 zu den seltenen Erkrankungen [3]. Meist manifestiert sich das GGS bereits im Jugendalter.

Diese autosomal-dominante Erkrankung wird durch eine Mutation im *PTCH1*-Gen auf Chromosom 9 ausgelöst [2, 3]. Das Gen kodiert den sog. „Patched-Rezeptor“, welcher ein negativer Regulator des Proteins „Sonic Hedgehog“ ist. „Sonic Hedgehog“ ist in der Embryonalentwicklung an multiplen Organentwicklungen sowie an der Zellproliferation postpartal beteiligt. Im ophthalmologischen Bereich erklärt dies nicht nur die zahlreich auftretenden Basalzellkarzinome (BCC), Plattenepithelkarzinome oder deren Vorläufer, sondern auch die embryonalen Fehlbildungen, die am Auge auftreten können.

Zur Diagnose des GGS wurden Kriterien (Tab. 1) formuliert. Laut den aktuellen Empfehlungen müssen 2 Hauptkriterien oder 1 Hauptkriterium sowie 2 Nebenkriterien zur Diagnosestellung vorliegen, nach denen bei unserer Patientin ein GGS vorlag. Die Diagnose sollte durch eine genetische Analyse gesichert werden.

**Table 1:** 

Hauptkriterien	Mehr als 2 Basalzellkarzinome (BCC) oder 1 unter 20 Jahren Histologisch gesicherte Kieferzysten 3 oder mehr palmare oder plantare Hyperkeratosen („Pits“) Ektopische intrakranielle Kalzifikationen Deformitäten der Rippen Familiäre Anamnese eines Gorlin-Goltz-Syndroms
Nebenkriterien	Makrozephalie Medulloblastom Andere Skelettanomalien (z. B. Syndaktylie, Polydaktylie, Anomalien der Wirbelkörper, Lippen- oder Gaumenspalte, Brückenbildung der Sella turcica) Augenanomalien (Katarakt, Kolobom, Mikrophthalmus) PTCH-Mutation Fibrome an Herz oder Ovarien Lippen-Kiefer-Gaumen-Spalte

Neben Keratinzysten, die bisher selten im Rahmen des GGS beschrieben wurden, ist auch die Myopie typisch für das GGS [5].

Die Keratinzysten rezidivieren häufig, wahrscheinlich weil die vollständige Exzision der Kapseln durch die derbe Verklebung mit dem Tarsus schwierig ist. Im Gegensatz zu Chalazien und Hordeola zeigt sich dabei keine Entzündungsreaktion um die Zysten, und die Zystenwand besteht aus verhornendem Plattenepithel [1]. Die Keratinzysten können auch spontan, wie bei unserer Patientin temporal der rechten Karunkel, verschwinden. Daher ist abhängig vom Leidensdruck der Patienten abzuwägen, ob die Zysten entlastet werden. Die Therapie der Keratinzysten erfolgt konservativ oder chirurgisch, jedoch nicht durch die sog. Hedgehog-Inhibitoren [4], die nur bei multiplen, nicht mehr operablen Basaliomen zum Einsatz kommen.

In der Literatur wird aufgrund der Rezidivhäufigkeit und der harten Kapsel der Keratinzysten eine En-bloc-Exzision mit zusätzlicher Tarsektomie oder alternativ eine ausgedehnte Resektion der Zyste mit tarsaler Elektrokoagulation der Kapsel empfohlen [1].

**Figure 3:**
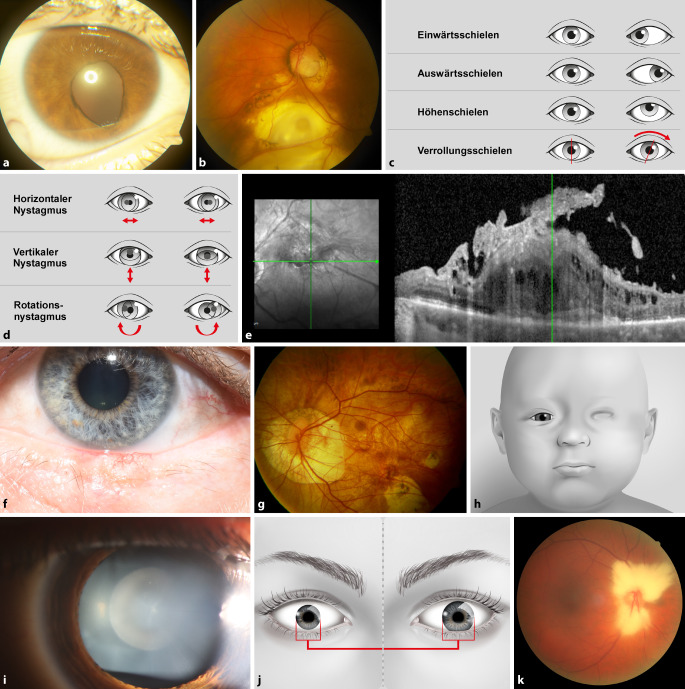

Neben einer Katarakt sind beim GGS nicht nur Kolobome an Iris, Netzhaut und Papille beschrieben, sondern auch vitreoretinale Interface-Erkrankungen, Myopie, myelinisierte Nervenfasern, Hypertelorismus sowie Strabismus mit Amblyopien, wahrscheinlich aufgrund von anatomischen Anomalien (Abb. 3; [3, 5]).

## Fazit für die Praxis

- Multiple Keratinzysten können neben Basalzellkarzinomen bei einem Gorlin-Goltz-Syndrom (GGS) vorkommen.
- Nicht immer muss ein Basalzellkarzinom bei GGS schon im jungen Erwachsenenalter vorliegen.
- Das GGS sollte aufgrund der multiplen Fehlbildungen mit variabler Expressivität interdisziplinär betreut werden. Aufgrund der multiplen Fehlbildungen wird die Diagnose meist schon im frühen Kindesalter gestellt.
- Palmare und plantare „Pits“ können durch intensives Anfeuchten quellen und besser visualisiert werden.
